# The burden of preventable hospitalizations before and after implementation of the health transformation plan in a hospital in west of Iran

**DOI:** 10.1017/S1463423618000841

**Published:** 2019-07-01

**Authors:** Bakhtiar Piroozi, Mohammad Amerzadeh, Hossein Safari, Amjad Mohamadi-Bolbanabad, Abdorrahim Afkhamzadeh, Yadolah Zarezadeh, Javad Mahmoudi, Sedigheh Salavati

**Affiliations:** 1 Assistant Professor in Health Policy, Social Determinants of Health Research Center, Research Institute for Health Development, Kurdistan University of Medical Sciences, Sanandaj, Iran; 2 Department of Health Management and Economics, School of Public Health, Tehran University of Medical Sciences, Tehran, Iran; 3 Assistant Professor in Health Policy, Health Promotion Research Center, Iran University of Medical Sciences, Tehran, Iran; 4 Assistant Professor in Health Services Management, Social Determinants of Health Research Center, Research Institute for Health Development, Kurdistan University of Medical Sciences, Sanandaj, Iran; 5 Associate Professor of Community Medicine, Social Determinants of Health Research Center, Kurdistan University of Medical Sciences, Sanandaj, Iran; 6 Associate Professor of Medical Education, Social Determinants of Health Research Center, Research Institute for Health Development, Kurdistan University of Medical Sciences, Sanandaj, Iran; 7 Bachelor of Medical Recording, Student Research Committee, Kurdistan University of Medical Sciences, Sanandaj, Iran; 8 Assistant Professor in Health Policy, Maragheh University of Medical Sciences, Maragheh, Iran

**Keywords:** ambulatory care, health expenditure, health system reform, hospitalization, primary health care

## Abstract

**Background:**

Increased number of preventable hospitalizations (PHs) for ambulatory care sensitive conditions (ACSCs) represents less efficiency and low access to outpatient and primary health care, leading to waste of health system resources.

**Aim:**

The purpose of this study is to assess the quality of outpatient and primary health care using the rate of PHs for ACSCs and to estimate the economic burden of ASCS before and after the implementation of the health transformation plan (HTP) in Iran.

**Methods:**

This research was a before–after quasi-experimental study. The study population included all patients hospitalized in the largest general hospital of Kurdistan province with five diseases such as asthma, diabetes, hypertension, congestive heart failure, and chronic obstructive pulmonary disease in 2014 (before the implementation of the HTP) and 2015 (after the implementation of the HTP). Data were analyzed by SPSS v.20 using Chi-square test.

**Findings:**

Total number of hospitalizations before and after the implementation of the HTP was 1501 and 1405, respectively. Moreover, the proportion of PHs in all types of the hospital admissions before and after the implementation of the HTP was 47% and 49%, respectively. There was no statistically significant difference between the number of PHs before and after the HTP. In total, PHs imposed 885 798 US$ and 9920 bed-days on health system before and after the implementation of the HTP.

**Conclusion:**

Despite the previous expectations of policy makers for improving quality, efficiency, and access to primary health care through implementation of the HTP, proportion of PHs is considerable and it imposes a lot of costs and bed-days on the health system both before and after the HTP.

## Introduction

Ambulatory care sensitive conditions (ACSCs) such as diabetes, hypertension, congestive heart failure, chronic obstructive pulmonary disease, and asthma are among the health problems which can be managed by timely and effective outpatient and primary health care (Rizza *et al.*, 2007; Purdy *et al.*, 2009). Preventable hospitalizations (PHs) for ACSCs is an indicator for assessing the quality and accessibility of ambulatory and primary health care services (Niti and Ng, 2003; Nedel *et al.*, 2008). Increasing financial access through expanding social protection schemes and social insurance along with expanding primary and outpatient care services funded through public resources can tackle the poor access to health care and low utilization of health services (Parchman and Culler, 1999; Roland *et al.*, 2005; Bottle *et al.*, 2006; Evans and Etienne, 2010; World Health Organization, 2010; Freund *et al.*, 2013).

## Iran’s health system

Health care delivery system in Iran has three levels. First level includes primary health care, which is funded by public sector and widespread healthcare network throughout the country. Such healthcare services are provided at rural level by health houses and rural health care centers and at urban level in urban health care centers. Second level services are provided by the public, semi-public, and private sector. Nevertheless, the public sector is dominant. In Iran, general health services are provided by universities of medical sciences. Universities of medical sciences are affiliated with the Ministry of Health and Medical Education. Each province has at least one university of medical sciences which provides health services to people through health houses, urban health centers, clinics, and hospitals. There are three main insurance schemes in Iran including health services insurance, social security, and armed forces. The health services insurance includes two funds for civil servants and rural residents. Rural health insurance covers residents of villages and towns with less than 20 000 population; all the premium of these people is paid by the government. The social insurance scheme also covers part of the civil servants, institutions, manufactories, etc. (Piroozi *et al.*, 2017).

In recent years, despite the relative success of providing primary health care at the first level as well as the interaction between this level and the subsequent levels of services provision in rural areas, referred to as the referral system, initial health care provision has been considered weak and non-systematic in urban areas. Incomplete coverage of insurance has also contributed to this problem, and it has limited the access of urban population to outpatient and specialist services (Rashidian *et al.*, 2013). According to *Iran’s* Multiple Indicator Demographic and Health *Survey* 2010 (DHS) conducted by the National Institute for Health Research, 17% of Iranian households were not covered by any health insurance (Rashidian *et al.*, 2012).

## Health transformation plan

In order to tackle health problems in Iran, Ministry of Health and Medical Education – as the main trustee of the health system – has initiated the health transformation plan (HTP) since May 15, 2014. The purpose of this plan is to move towards Universal Health Coverage and improve equity and quality in access to health services. This plan includes diverse packages and interventions, some of which are expected to increase, directly or indirectly, access to primary health care and outpatient services and, subsequently, reduce the number of PHs for ACSCs. These interventions include:

- Covering people without basic health insurance (about 17% of the population) by Iranian health insurance for free.
- Facilitating physicians’ retention in deprived areas along with incentive payments for increasing their availability in health services.
- Improving the quality of outpatient care services in university hospitals by changing the behavior of physicians and standardizing the duration of each outpatient visit to improve the quality of outpatient care services, increasing the incentives of service providers, and by keeping physicians in the university sector.
- Strengthening primary health care in rural areas and towns with less than 20 000 population by raising the budget of the rural family physician plan and by increasing the recruitment of physicians, midwives, and other health care personnel along with renovating and equipping the rural health centers.
- Provision of basic health care for a population of 8.5–10 million people living in 700 outskirts of urban areas through a health-care network, which have no access to primary health care.
- Creating health awareness and strengthening the national self-care plan (Piroozi *et al.*, 2017).

Now, we intend to answer two main questions in this study: Did the packages and interventions implemented through the HTP resulted in significant decrease of PHs percentage? And what is the amount of imposed costs and bed-days due to PHs on the health system before and after the implementation of the HTP?

This is the first study in Iran which is conducted to show the economic burden of PHs and its differences before and after the HTP.

## Methods

This was a before and after quasi-experimental study. In order to conduct this study, we needed to choose a province that was not a referral hub for referring patients from other provinces of the country. Kurdistan province had this condition. Kurdistan – as one of the deprived provinces of Iran – is located in west of Iran, with 8 cities and 14 hospitals (Moradi *et al.*, 2017; 2018). Study population was equal to the sample size and included all patients hospitalized due to one of the five diseases such as asthma, diabetes, hypertension, congestive heart failure, and chronic obstructive pulmonary in January, February, and March of 2014 (one year before the implementation of the HTP) and 2015 (one year after the implementation of the HTP) in Tohid General Hospital affiliated to Kurdistan University of Medical Sciences as the largest hospital in the province. It is necessary to mention that the total number of hospitalizations in the three-month period related to one year before and after the HTP was 12 002 and 13 241, respectively.

These five diseases are among the most common noncommunicable diseases in Iran and responsible for frequent hospitalizations (Rezazadeh Kermani, 2008; Esteghamati *et al.*, 2009; Varmaghani *et al.*, 2016; Peimani *et al.*, 2017). Tohid Hospital is located in Sanandaj as the center and largest city in the province with a population of over 600 000 (SCI, 2017). The diagnostic codes of the hospitalized patients were extracted from the hospital information system (HIS) during the study, and patients, whose main reasons for hospitalization were ‘Preventable hospitalizations for ambulatory care sensitive conditions (ACSCs)’, were identified. In HIS, the diagnostic codes were based on International Statistical Classification of Diseases (ICD)-10. Also, the gender, location, bill costs (total hospitalization costs), type of insurance, and bed-days were extracted from HIS for each hospitalization.

Many countries have defined a list of PHs for ACSCs, but these codes have not been defined for Iran yet. Therefore, we prepared a list of these codes based on the literature review, and, then, views of a professional group including some physicians and primary health care experts were asked about the codes through holding common meetings. Finally, an agreement was made on a list of PHs (Table 1) (Niti *et al.*, 2003; Rizza *et al.*, 2007; Purdy *et al.*, 2009; Freund *et al.*, 2013). Costs were reported in US dollars based on the mean of official exchange rate of Central Bank of Iran in each period. Data were described and analyzed by SPSS v.20 software using frequency, percentage, mean, and Chi-square test.

**Table 1.** List of ICD-10 codes for preventable hospitalizations in five diagnostic groups

ID	Ambulatory care sensitive condition	ICD-10 codes
1	Asthma	J45, J46
2	Congestive heart failure	I11.0, I50, J81, I130 I255
3	Chronic obstructive pulmonary disease	J10.0, J11.0, J12, J13, J14, J15, J16, J18, J20, J21, J22, J41, J42, J43, J44, J47
4	Diabetes complications	E10.0-E10.8, E11.0- E11.8, E12.0- E12.8, E13.0- E13.8, E14.0- E14.8, E139, E149
5	Hypertension	I10, I10.0, I10.1, I11, I11.9

## Results

Table 2 presents burden of PHs before and after the HTP in the five diagnostic groups. In 2014, out of 1501 hospitalizations for the five diagnostic groups, 47% (760 hospitalizations) were preventable. Of them, 82% of the hospitalizations in the Hypertension group and 75.6% of all hospitalizations in Asthma group were preventable. In 2015, out of 1405 hospitalizations for the five disease groups, 49% (678 hospitalizations) were preventable, of which 85% and 73.4% of all hospitalizations in the Hypertension and Asthma groups were preventable, respectively.

**Table 2.** Statistics of preventable hospitalizations before and after the health transformation plan (HTP) in Tohid Hospital

Disease category		Number (%) of hospitalizations before HTP	Number (%) of hospitalizations after HTP	χ^2^	*P*-value
Asthma	Preventable	90 (75.6)	80 (73.4)	0.15	0.699
	All hospitalization	119	109		
Congestive heart failure	Preventable	507 (69)	431 (66)	1.3	0.253
	All hospitalizations	734	652		
Chronic obstructive pulmonary disease	Preventable	6 (1.7)	11 (3)	1.36	0.243
	All hospitalizations	348	358		
Hypertension	Preventable	144 (82.2)	142 (85)	0.47	0.439
	All hospitalizations	175	167		
Diabetes complications	Preventable	13 (10.4)	14 (11.8)	0.132	0.717
	All hospitalizations	125	118		
Total	Preventable	760 (47)	678 (49)	1.64	0.201
	All hospitalizations	1501	1405		

Based on the *χ*^2^ test, there was no significant difference between the number of PHs before and after the implementation of the HTP (*P* > 0.05).

Some characteristics of PHs are presented in Table 3. Out of 760 PHs for the five disease groups in 2014, 54.9% (417) were women, 73% (555 people) were urban residents, 99.6% (757) had basic insurance, and 3.6% (27) had complementary insurance. In 2015, out of 678 PHs, 53.7% (364) were women, 66.8% (453 people) were urban residents, 99.7% (676) had basic insurance, and 7.1% (48) had complementary insurance. The mean age of people with PHs was 64.25 (SD: 15.47) years old in 2014 and 63.66 (SD: 15.6) years old in 2015.

**Table 3.** Preventable hospitalizations characteristics before and after health transformation plan (HTP)

Variables		Preventable hospitalizations for five causes		χ^2^	*P*-value
		Before HTP number (%)	After HTP number (%)		
Sex	Male	343 (45.1)	314 (46.3)	0.201	0.341
	Female	417 (54.9)	364 (53.7)		
Residence	Urban	555 (73)	453 (66.8)	6.59	0.01
	Rural	205 (27)	225 (33.2)		
Basic insurance	Have	757 (99.6)	676 (99.7)	0.997	0.608
	Do not have	3 (0.4)	2 (0.3)		
Complementary insurance	Have	27 (3.6)	48 (7.1)	9.01	0.003
	Do not have	733 (96.4)	733 (96.4)		

Five causes include asthma, congestive heart failure, chronic obstructive pulmonary disease, hypertension, and diabetes complications.

As Table 4 shows, the average patients’ payment for the five diagnostic groups was 50.13 US$ in 2014 and 34.77 US$ in 2015, which fell by 30% in 2015. The average number of total hospital admissions costs for these five disease groups for 2014 and 2015 was 374.36 US$ and 795.85 US$, respectively, which shows an increase of 112% in 2015 compared to the previous year (Table 4).

**Table 4.** Average patient payments and total hospitalization costs for preventable hospitalization before and after the health transformation plan (HTP) in US dollar

Variables		Before HTP		After HTP	
		Average	Total	Average	Total
Asthma	Patient payments	40.16	3655.36	26.52	2124.10
	Hospitalization costs	309.83	27 877.28	509.22	40 739.14
Congestive heart failure	Patient payments	51.58	25 230.56	38.39	16 286.60
	Hospitalization costs	377.53	191 416.73	786.67	33 9060.25
Chronic obstructive pulmonary disease	Patient payments	217.45	1304.80	41.17	452.95
	Hospitalization costs	1128.53	6760.28	795.34	8748.79
Hypertension	Patient payments	53.78	7373.14	29.28	4100.55
	Hospitalization costs	372.23	53601.57	953.24	135 359.60
Diabetes complications	Patient payments	61.99	806.32	43.70	611.93
	Hospitalization costs	372.55	4843.09	1119.77	15 676.41
Total	Patient payments	50.13	38 128.96	34.77	23 576.12
	Hospitalization costs	374.36	284 508.95	795.85	539 584.18

Results showed that the average length of stay for PHs in 2014 and 2015 was 6.75 and 7.06 days, respectively, which increased by 4.6% from 2014 to 2015. Total hospital stay for PHs was 5134 days in 2014 and 4784 days in 2015 (Table 5).

**Table 5.** Average and total patients’ bed-days for preventable hospitalization before and after implementation of health transformation plan (HTP)

		Bed-days	
		Before HTP	After HTP
Asthma	Average bed-days	6.97	6.98
	Total bed-days	636	558
Congestive heart failure	Average bed-days	7.01	7.34
	Total bed-days	3557	3163
Chronic obstructive pulmonary disease	Average bed-days	9.5	5
	Total bed-days	57	88
Hypertension	Average bed-days	5.63	6.1
	Total bed-days	811	871
Diabetes complications	Average bed-days	5.6	7.57
	Total bed-days	73	106
Total preventable hospitalization	Average bed-days	7.06	6.75
	Total bed-days	5134	4786

## Discussion

Based on the findings of the current study, about half of the hospitalizations before and after the implementation of the HTP was considered to be preventable and had imposed a total of 25 592 665 US$ and 4786 bed-days on health system. This indicates a high percentage of PHs and costs caused by them in Iran.

Results of a national study in Singapore showed that 6.7% of all hospitalizations were considered preventable for ACSCs for five diseases such as asthma, congestive heart failure, chronic obstructive pulmonary disease, diabetes mellitus, and hypertension between 1991 and 1998 (Niti *et al.*, 2003). A hospital level study in Italy also showed that 31.5% of total hospital admissions were preventable for the same five diseases as mentioned in our study. In this study, the odd ratio of hospitalization due to ACSCs was reduced by increasing access to and utilization of outpatient health services (Rizza *et al.*, 2007).

A study conducted in the UK using national data for 36 groups of diseases showed that the burden of PHs because of ACSCs on the NHS system was 1.9 million hospital admissions, 16.6 million bed-days, and 2.9 billions of pounds in 2005 and 2006 (Purdy *et al.*, 2009). It seems that the percentage of PHs in Iran is higher than that of other countries, which imposes high costs and bed-days on Iranian health system.

Based on the results of our study, there was no statistically significant decrease or increase in the total number of hospital admissions and PHs after the implementation of the HTP compared with before its implementation. The result of the current study is different from the previous studies which claimed that extending access to outpatient care and primary care would reduce the burden of PHs (Lindström *et al.*, 2003; Dafny and Gruber, 2005; Laditka *et al.*, 2005; Ionescu-Ittu *et al.*, 2007; Rizza *et al.*, 2007). In a study in Iran, there was no significant difference between the rate of PHs before and after the family physician and rural insurance plan based on the Chi-square test (Salavati and Rashidian, 2017). In another study in Ireland, free access of people to urban family physicians over seventy years did not affect the burden of PHs (Nolan, 2011). However, in a study in Brazil, coverage of the family health plan for PHs had a protective effect (Carvalho *et al.*, 2015). Also, in a study in London, the provision of diabetes care at primary care level resulted in a reduction in the burden of PHs (Saxena *et al.*, 2006). Furthermore, in a study in Sweden, there was a reverse relationship between the number of general practitioners’ visits and the rate of PHs (Kohnke and Zielinski, 2017). In addition, another study in the United States showed that not only insurance coverage but also structural changes in outpatient and primary health care, especially in deprived areas, are essential to reduce PHs. Another study also found that PHs increased with increasing insurance coverage for noninsured individuals (Friedman and Basu, 2001).

In our study, in spite of an increase in the insurance coverage in Kurdistan province from less than 80% before the implementation of the HTP to 99% after it, there was no reduction in the burden of PHs. Thus, it seems that only an increase in the population coverage cannot be sufficient to reduce the burden of PHs. Therefore, services packages must be reviewed and some other interventions should be considered to promote the efficiency of provided health care services in health plans.

Despite the fact that before the implementation of the HTP more than 20% of the Kurdistan province was not covered by any insurance, 99.6% of hospitalized patients had a basic health insurance before the implementation of the HTP. It shows that people without health insurance utilized hospitalization services less than those with insurance (Piroozi *et al.*, 2017). In a number of studies, the impact of continuity on PHs has been measured as one of the features of primary health care, and their results indicated the negative effect of the continuity of primary care on burden of PHs (Menec *et al.*, 2006; Ionescu-Ittu *et al.*, 2007).

Based on our findings, despite a significant increase in the average hospitalization costs, there was a significant decrease in the average direct payment of the hospitalized patients after the HTP compared to before its implementation. This decline is due to the implementation of the package ‘Reducing payment rates for hospitalized patients in university hospitals’ in the form of HTP. According to this package, hospitalized patients who live in cities are required to pay a maximum of 6% of hospitalization costs, and patients who live in villages and cities under 20 000 population, if referred through the referral system, are required to pay a maximum of 3% of total hospitalization costs (Piroozi *et al.*, 2017). An increase in the hospitalization costs is due to the introduction of a new book with updated relative value units, which resulted in using real and high medical tariffs.

There is another study conducted in Iran which confirms the findings of our study; in spite of an increase in total hospitalization costs, out of pocket payments of hospitalized patients decreased significantly after the implementation of the HTP compared to before its implementation (Piroozi *et al.*, 2017).

## Limitations

Although the studied hospital was the largest one in the province, its results cannot be generalized to the whole country. In addition, in this study, we assessed the differences of PHs percentage before and after the HTP immediately after the reform; thus it will be needed to evaluate the impact of HTP on PHs in a long time period.

## Conclusion

Despite the previous expectations of policy makers for improving the quality, efficiency, and access to primary health care through the implementation of the HTP, there were no significant differences between percentage of PHs before and after the HTP. The rate of PHs was very high both before and after the HTP, and it imposed high costs and bed-days on the health system. It seems that there is a serious need to design and implement some interventions to improve quality and utilization of outpatient and primary health care services.
